# Second Life for Recycled Concrete and Other Construction and Demolition Waste in Mortars for Masonry: Full Scope of Material Properties, Performance, and Environmental Aspects

**DOI:** 10.3390/ma17205118

**Published:** 2024-10-19

**Authors:** Vadim Grigorjev, Miguel Azenha, Nele De Belie

**Affiliations:** 1 Magnel-Vandepitte Laboratory, Department of Structural Engineering and Building Materials, Faculty of Engineering and Architecture, Ghent University, Technologiepark Zwijnaarde 60, 9052 Gent, Belgium; 2 ISISE, Department of Civil Engineering, University of Minho, Building 02, 4800-058 Guimarães, Portugal

**Keywords:** construction and demolition waste, fine recycled aggregates, masonry mortars, renders, plasters, cultural heritage, sustainability

## Abstract

This review presents the scope of current efforts to utilize recycled construction and demolition waste in mortars for masonry. More than 100 articles are divided into groups pertaining to the type of mortar, different binder systems, the type of construction and demolition waste (CDW), and its utilization specifics. Cement-based mortars dominate this research domain, whereas recycled concrete is the main material employed to replace virgin aggregates, followed by recycled masonry and recycled mixed waste aggregates. Such application in cement-based mortars could increase water demand by 20–34% and reduce strength by 11–50%, with recycled concrete aggregates being the most favorable. Natural aggregate substitution is disadvantageous in strong mortars, whereas weaker ones, such as lime-based mortars, could benefit from this incorporation. The extent of this topic also suggests possibilities for different recycled material use cases in mortars for masonry, although the available literature is largely insufficient to infer meaningful trends. Nonetheless, the most relevant knowledge synthesized in this review offers promising and environment-conscious utilization pathways for recycled concrete and other construction and demolition waste, which brings opportunities for further research on their use in mortars for masonry and industrial-scale applications.

## 1. Background

### 1.1. Preamble

The move towards a more sustainable future involves contributions from the largest industries, with the building industry no exception. Construction business is reportedly responsible for 37% [1] of anthropogenic carbon dioxide emissions, which, despite being eye-opening, could be expected when construction, maintenance, and demolition processes are considered through the complete life cycle of modern structures and infrastructure. There are, however, multiple examples of impactful changes that could be implemented to reduce the adverse effects this industry bears. Alternative energy sources, resource and logistical efficiency, new materials, and reuse and recycling of various waste streams are some of the possibilities being researched, evaluated, and applied currently. This work is focused on the evaluation of the progress and existing challenges in the area of recycling construction and demolition waste generated after new construction and produced when demolishing old structures, respectively.

Even though construction and demolition waste has existed ever since construction emerged on a large scale in human history, the endorsement of recycling is still relatively young. The big reason is the inevitable dependence on definitions, implementation policies, and regulations [2,3,4,5]. Despite that, there have always been multiple possible pathways for CDW reintroduction into new construction projects. With concrete being the most consumed man-made material on the planet, it became a primary target for the application of waste materials, especially selectively demolished concrete aggregates. Infrastructure applications, particularly road construction, have also long been the recipients of CDW rubble, especially that of mixed origin or containing bituminous materials [6,7,8].

Numerous research articles have presented results uncovering various potential applications for recycled CDW that come in the physical form of coarse aggregates [9,10,11]. Fine recycled aggregates, on the other hand, have received less attention from researchers, the industry, and the legislators, as this fraction is considered to be a by-product of coarse recycled aggregate production and is often associated with undesirable properties of recycled materials, thus hindering more widespread adoption. Nonetheless, this notion is rapidly changing in research, as the drive to enhance the full recycling potential of waste materials to facilitate the transition into a circular economy has resulted in an annually growing number of publications discussing various applications of fine recycled CDW, with some notable examples being the works of Winkler and Müeller [12], Evangelista and de Brito [13], Zega and Di Maio [14], and Zhao et al. [15].

There is a strong research concentration regarding the application of fine recycled CDW in concrete. Due to the high volume of publications, some authors have synthesized the knowledge base in review articles [16,17,18], promoting the dissemination of the state of the art in this area. However, other use cases for cementitious materials, which require particular considerations both in research and industrial practice, could benefit from separate analyses. One such use case is mortars for masonry construction.

The focus on potential building waste applications in masonry structures is increasingly more prominent in research starting from the 21st century. However, the real cases where recycled materials have been used in new masonry construction are limited. To address this gap, some work has been carried out to evaluate the feasibility of using such materials for the creation of new building blocks [19,20,21]. However, the main focus of this review is on the other component of masonry—the mortar. Specific aspects of construction and demolition waste recycling through masonry mortars and renders have been explored previously by a dedicated systematic review and meta-analysis based on the performance of mortars [22]. Other reviews on the topic have been presented as well [23,24], whilst some broader review works have also included mortars for masonry in their breadth to a variable extent [25].

Although previous reviews have provided comprehensive summaries and analyzed the data available at their respective times, the continuous and expanding research efforts, along with the increasing variety of recycled materials and their utilization opportunities in mortars for masonry, necessitate an updated review. This study aims to deliver a meticulously categorized and exhaustive overview of the latest research findings in the fields of CDW recycling and masonry construction, thereby facilitating the ongoing transition towards a more sustainable built environment.

### 1.2. Relevance

Admittedly, periodic reviews of published literature are beneficial in both summarizing the ongoing research trends and shaping future directions, along with the inevitable identification of undersaturated areas. In this light, a new scoping review is presented with the main focus on the peculiarities of CDW incorporation in mortars for masonry. This review allows us to identify the areas of high interest and/or research gaps whilst reducing the ambivalence of published work.

Even though the primary sources of data gathered for this review are academic works, its significance could transcend academia to the industrial level, with relevant conclusions for masonry mortar producers, binder materials producers, and contractors, and also be relevant to the national and international regulatory bodies.

In particular, special care is taken to distinguish between the different types of applications of mortars for masonry. Naturally, the role of a mortar in masonry construction differs according to the construction system, but there are further intricacies arising from the variability of materials and their proportions, inaccuracies of the testing methods, and comparability of the results obtained in a laboratory with those on building sites. In addition to the prevalent challenges from societal, economic, regulatory, and environmental perspectives, the multifariousness of the topic on a research level has also contributed to a lack of interest from the industry [26,27,28,29], and addressing this issue is one of the main motivational points of this review.

### 1.3. Type of Review

This review [30] is designed to present the scope of the topic in the following ways:

- Summarizing available evidence on the use of recycled construction and demolition waste in mortars for masonry applications;
- Grouping said evidence into well-structured categories based on main differentiating factors;
- Exploring the possible ambiguity in terminology, research methods, results, and conclusions;
- Identifying broadly researched specific parts of the main theme, suitable for systematic reviewing and analysis;
- Identifying the research questions that require further deliberations;
- Providing an informational basis for future research and decision-making.

With regard to the particular topic in question, when ample data on a specific part of it are present in conducted research, it is analyzed in terms of the most prominently evaluated material properties, with a strong focus on those included in relevant standards.

### 1.4. Study Selection Criteria

To aid the aforementioned tasks, the research works were collected primarily using the Web of Science (WoS) [31]. Indexed articles were searched for using the following words appearing in the topic (title, keywords, and abstract):
masonry render * plaster * historic * repairANDmortar *ANDconstructioni.e.,demolitionrendering mortars with recycled aggregateswasteplastering mortar with construction and demolition wasteCDWrecycle *aggregate *historic mortars with CDW* allows for differently phrased word endings.

Based on this search, more than 800 publications were found. These were further screened to select the articles focusing on mortars that contain recycled CDW materials and are specifically related to masonry construction, as opposed to concrete construction, which dominates the research domain.

This review is based on three selected types of mortars used in masonry construction: masonry mortars, rendering and plastering mortars, and historic/repair mortars. The first category is defined as per EN 998-2 [32] and includes mortars for structural and non-structural bedding, jointing, and pointing of masonry, i.e., mortars for laying brick and block and finishing the joints in masonry walls. Renders and plasters are exclusively coating mortars, with the former being applied on external or environment-exposed walls and the latter—used for interior works. These types are subjected to the requirements of EN 998-1 [33]. In European terminology, bedding and coating mortars are referred to as “mortars for masonry”, and they are tested according to the EN 1015 [34] series of standards. Finally, cultural heritage mortars, also known as historic mortars (or repair/renovation mortars), are bedding, pointing, and coating mortars intended specifically for the conservation and repair of historic structures. These are described in EN 16572 [35], and some requirements for such mortars are presented in ASTM C1713 [36].

By applying the above-mentioned criteria, around 100 studies were selected as suitable for this scoping review. Throughout this work, all mortars are collectively called “mortars for masonry”, i.e., deviating from the EN 998 definition by including the cultural heritage mortars under this umbrella term.

### 1.5. Research Themes and Review Structure

**Figure 1:**
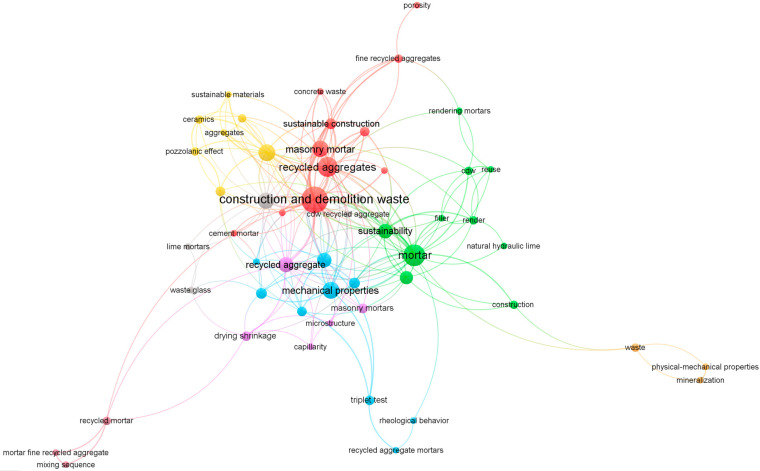
Selected research keyword map. Different colours represent keyword clusters and their interconnectivity.

With due consideration for the different types of masonry-related applications, the seemingly simple topic of mortars with recycled materials is expanded tremendously by further separation into smaller parts. That can be witnessed through the spread of the keywords and various links between them, as shown in Figure 1, visualized using VOSviewer 1.6.19 [37]. Naturally, the majority of the research revolves around the use of CDW, as set during the study selection stage. The following topics explored in the research are clustered around mortars, their properties, recycled aggregates, and sustainability. Evidently, there are also more niche aspects of the current knowledge base, such as studies on particular waste streams and specific preparation and testing procedures, as demonstrated by the weaker links to other research aspects in the visualized representation of common keywords in Figure 1.

**Figure 2:**
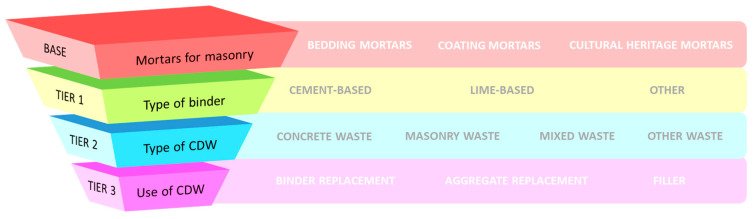
Tier-based structure of selected studies.

To address the complexity of the topic, a tier-based system has been utilized logically, joining the two main materials of interest: mortars and construction and demolition waste—Figure 2.

The first tier of the review divides mortars based on their binding materials. The choice of such grouping is justified by the higher impact binders might have on fresh and hardened mortar properties compared to the other dry constituent—aggregates. The main binders encountered in literature are as follows:

- Cement—various types;
- Lime—various types, either in single binder mortars or in combination with other binders;
- Others, such as case-specific materials, commercial binders of known or unknown composition (such as masonry cement), alkali-activated materials, different SCMs, and their combinations.

The second tier addresses the differences in construction and demolition waste materials. It concerns the type of CDW according to its composition, namely subdivided into the following groups:

- Recycled concrete;
- Recycled masonry—in this review, an umbrella term for bricks, tiles, and ceramics that also includes any types of masonry blocks and naturally, any remainders of mortars, renders, and plasters;
- Recycled mixed waste—normally unsorted, a mix of the two aforementioned types and possibly traces of metals, glass, soil, plastics, wood, etc.;
- Others—specific CDW, such as insulation materials or sanitary ceramics waste.

The final tier combines both subjects of mortars for masonry and waste materials by highlighting the different utilization cases of the latter. In this regard, waste materials can be used in mortars by (partial) replacement of the binders and (partial) replacement of the aggregates or as fillers. The latter option includes recycled fine material (typically <250 µm particle size) in addition to mortar mixes and partial aggregate fraction replacement, essentially increasing the fineness or substitution of the corresponding finest aggregates. Due to distinct considerations in current research and different effects on mortar performance, the use of recycled CDW as filler is presented as a separate category in the final tier.

The described tier-based structure allows for a streamlined assessment of the research scope and a simple synthesis of performance evaluation for specific mortars utilizing particular CDW. The performance is assessed based on the relevant fresh and hardened state mortar properties with a presentation of the most important factors, i.e., strength classes of masonry mortars. Furthermore, particular care is taken to present the significance of masonry-scale properties, which in most cases relate to the mortar–substrate interface. The mechanical properties at this level are frequently and interchangeably termed as “bond strength” and “adhesive strength”, although for the purpose of this review and in line with the current European standards, these terms are separated into the following:

- Bond strength—initial shear strength of the mortar–brick interface when mortars are used for bedding purposes, tested in accordance with EN 1052-3 [38] or similar methods.
- Adhesive strength—the strength of the coating mortar layer, i.e., render or plaster, when tested in accordance with EN 1015-12 [39] or similar methods.

A narrative overview approach is taken for the lowest tiers containing less than five relevant studies, presenting difficulties in reasonable data analysis. Extra attention is given to the environmental performance results in studies that have considered this, as the whole topic stems from the premise of sustainable development.

## 2. Current Knowledge Base

### 2.1. Cement-Based Binder

Different variations in cement, based on EN 197-1 [40] and other regional standards, are the most commonly used binder materials for masonry mortar applications. Unsurprisingly, the majority of studies considered the use of cement with clinker substituted by supplementary cementitious materials, which is in line with current developments towards sustainability in the built environment [41].

#### 2.1.1. Cement—Concrete CDW

Cement has been used extensively as the main binding material in concrete worldwide, which implies that waste generated during building and end-of-life demolition stages of concrete structures is significant [42]. Admittedly, recycling concrete for various secondary use cases has been well researched, and the use of recycled concrete aggregates for masonry applications has also been studied rather extensively.

##### Cement—Concrete CDW—Binder Replacement

Replacement of the binder in cementitious systems can be associated with the highest benefits regarding their environmental performance [43]. However, there is a lack of research on masonry mortar binders being replaced by recycled concrete, i.e., in a powdered form. In fact, such a scenario was only explored recently by Ohemeng et al. [44]. This experimental campaign was set to utilize waste concrete collected from the demolition sites in South Africa, crushed and milled in a laboratory as a replacement of Portland cement (PC) (CEM I 52.5N) at levels of 15%, 30%, 40%, 60%, 75%, and 100%, by mass. Masonry mortars were then made using this combined binder in a 1:3 binder to aggregate ratio by mass. At the same level of mixing water, mortars with an increasing proportion of waste concrete powder have shown a reduction in flowability, an increase in setting time, and a small decrease in bulk density in a fresh state. On the other hand, the hardened properties have seen a drastic decline in mechanical strength at higher replacement levels, a reduction in the dry bulk density, and an increase in the water uptake, influenced by the higher porosity. Interestingly, the drying shrinkage of masonry mortars decreased with the incremental addition of the waste concrete powder, which could have been influenced by the reduced formation of hydration products and, therefore, a lower drying rate, as explained by the authors. The conclusions of this study revealed that 40–75% replacement of cement by waste concrete powder is the best option in terms of mortar properties, their cost, and environmental performance. A follow-up study by the authors [45] further improved in these aspects by showcasing even higher environmental and cost benefits by utilizing a binder composed of 60% cement powder and a combination of PC and Fly ash.

##### Cement—Concrete CDW—Aggregate Replacement

Natural aggregate replacement in masonry mortars using recycled concrete has been found to be the dominant category within this review. The main studies with short descriptions of the main details are presented in Table 1.

Data analysis was performed in order to qualitatively assess the effect of using concrete CDW in place of the aggregates in masonry mortars. Some of the most prominent properties of fresh and hardened mortars are used for benchmarking recycled concrete aggregate mortars against reference natural aggregate ones. The performance descriptors are presented in Table 2 for fresh characteristics and Table 3 for hardened properties. Some studies included few of the selected properties, whilst others were more complete, hence the differences in numbers. Each study was taken as one unit in this representation unless the results were mixed, i.e., better, similar, and inferior performance is achieved depending on the amount of recycled concrete aggregates in the mix. Further information regarding these properties is available in Supplementary Materials.

**Figure 3:**
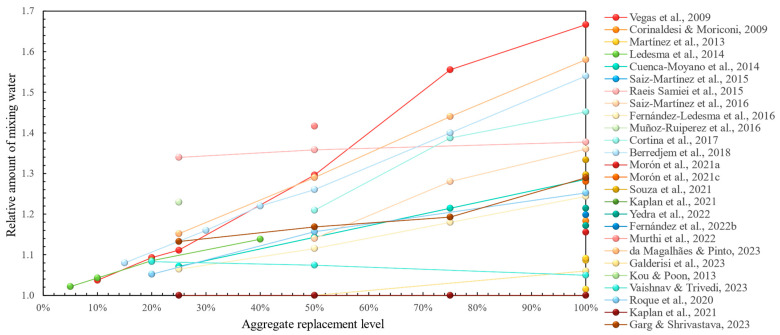
Initial mixing water requirement in recycled concrete aggregate mortars vs. replacement levels [46,47,48,49,50,51,52,53,54,55,56,57,58,59,61,62,63,64,65,66,67,68,69,70,72,73].

In terms of fresh properties, the majority of research has demonstrated that the use of recycled concrete aggregates inevitably leads to a higher mixing water requirement [46,47,48,49,50,51,52,53,54,55,56,57,58,59,60,61,62,64,65,66,67,68,69,71,72,73]. This is demonstrated graphically in Figure 3 with a clearly increasing trend of mixing water with aggregate replacement ratio. Fresh bulk density was shown to be lower in most studies, which in this case is considered positive due to weight savings in a structure, without considerations for any possible reductions in mechanical performance stemming from it. In terms of the air content, most studies did not demonstrate significant deviations from the reference [46,49,57,58], whereas, in [50,55], it was increased due to recycled concrete aggregate incorporation, which in this case was regarded as a positive feature due to the possibly increased workability of the fresh mortar. Some less-studied fresh properties, such as water retention, were improved [46,48,62,71], whilst workable life was shown to decrease with the addition of recycled aggregates [49,53,54], except in [73].

The properties of fresh mortar have not been extensively studied, and existing information is limited to a few standardized tests. This lack of comprehensive data is pivotal since the execution of masonry construction relies heavily on human input, making the fresh state of mortar critically important during the building stage.

**Figure 4:**
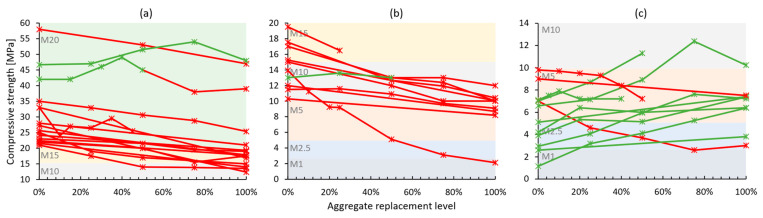
Compressive strength of recycled concrete aggregate mortars vs. replacement levels: (**a**) M20 category reference mortars; (**b**) M15 and M10 category reference mortars; and (**c**) M1–M5 category reference mortars. Green lines and markers indicate similar or better performance compared to reference mortars, and red indicates worse performance.

Hardened properties of cement-based recycled concrete aggregate mortars present larger variability in results across conducted studies—Table 3. Mechanical strength, which was within the experimental campaign of most works, was usually negatively impacted by the use of recycled aggregates [46,47,48,50,51,52,53,54,55,56,59,60,61,64,65,66,67,68,70]. Despite this, a significant portion of the mortar formulations had benefited from the natural aggregate substitution [49,57,58,62,63,69,71,72,73]. This is illustrated through compressive strength results in Figure 4. Based on the relevant standards [32,33], compressive strength is one of the most important properties describing both a bedding mortar and a coating mortar. For simplicity, all reviewed mortars were grouped following the European masonry mortar strength classes [32], i.e., M5, meaning a minimum of 5 MPa of compressive strength when tested at 28 days of age. The majority of stronger (at least M10 in reference formulation—Figure 4a,b) studied mortars suffered from the addition of recycled concrete aggregates, whereas for weaker mortars (Figure 4c), the result was largely the opposite. Weak cement-based mortars are formulated with significantly lower binder content, which upscales the role aggregates have in strength development, and due to their heterogeneous physical properties, recycled aggregates could have presented a better particle packing or added to the overall hydration product formation.

With respect to other hardened mortar properties, some further trends could be established: the addition of recycled concrete aggregates resulted in a lower bulk density, higher capillary water absorption, and overall higher shrinkage.

**Figure 5:**
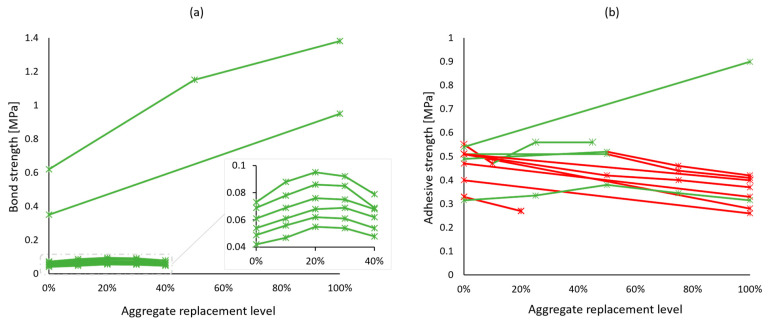
Bond strength (**a**) and adhesive strength (**b**) of recycled concrete aggregate mortars vs. replacement levels. Green lines and markers indicate similar or better performance compared to reference mortars, and red indicates worse performance.

Most importantly, hardened mortar performance should be assessed through application-related tests. For masonry mortars, the bond between the mortar and the bricks or blocks is crucial, yet only a few studies [47,66,70] have tested it through the evaluation of initial shear strength according to EN 1052-3 [38], although it was overall in favor of adding recycled concrete aggregates—Figure 5a. Other works have also considered the adhesive bond strength of mortars, which is an integral property of renders and plasters. More results pointed towards the inferior performance of recycled aggregate mortars in this regard (Figure 5b). Water vapor permeability—another property of a coating mortar—was found to increase with natural aggregate replacement [46,58,72]. This could be viewed both as a positive or a negative aspect, depending on the functional requirements of the coating.

Interestingly, the adhesive strength of renders and plasters was included in studies on masonry mortars [48,51,53,56] and vice versa when shear bond strength was evaluated in a study dedicated to coating mortars [70].

##### Cement—Concrete CDW—Filler

The use of recycled concrete fines as filler for masonry mortars and renders has been investigated by Braga et al. [74,75] and Jesus et al. [76,77]. These studies dealt with the incorporation of fines (<150 µm) from recycled concrete, obtained by sieving out the coarse fraction entirely. The mortars used in [74,76] were formulated with a volumetric ratio of 1:4 for cement and aggregates, while recycled concrete filler substituted natural sand in volumetric concentrations of 5%, 10%, 15%, and 20%. Follow-up studies on coating mortars further explored the effect of varying binder:aggregate ratios from 1:4 to 1:5 and 1:6 [75,77]. This incorporation of recycled fines resulted in improved workability, thus reducing the w/c ratios required to achieve the same consistency of mortar. Furthermore, the mechanical strength of these mortars increased compared to the reference mortars containing only natural aggregates, and more importantly, the adhesive properties of mortars were also enhanced by the presence of recycled concrete filler. Some inferior performance for capillary water absorption, shrinkage, and water vapor permeability was not significant enough to prevent authors from deeming the addition of recycled concrete waste fines a successful effort in reducing the environmental impacts of cementitious mortars.

#### 2.1.2. Cement—Masonry and Ceramic CDW

Masonry construction and demolition waste is not only limited to bricks and blocks with adhering mortar and render/plaster. Due to the abundance of different building blocks on the modern market as well as a plethora of material compositions in various masonry-related products, this review combines various ceramics, tiles, and other clay-based materials with general masonry waste as part of masonry and ceramic waste umbrella term.

##### Cement—Masonry and Ceramic CDW—Binder Replacement

One of the first attempts at cement substitution by ceramic waste has been documented by Corinaldesi et al. [78]. This work explored the 30% substitution of CEM II by red clay brick powder (<90 µm) in 1:3 cement:aggregate ratio mortars. These mortars were also supplemented by stainless steel and polypropylene fibers and compared against a similar formulation, which included 100% natural aggregate replacement by recycled brick aggregates. In terms of mechanical strength, the best performance was observed in recycled brick powder mortars after 90 days, likely due to the added pozzolanic effect. However, these mortars did not perform as well in the bond strength testing when compared to mortars without cement replacement, containing only recycled brick aggregates. A subsequent study has aimed to tackle this problem [79], albeit using different bricks. The mortar containing powdered brick as cement substitution had the same composition as in the above-mentioned study, but in this case, it was compared to a cement-based natural aggregate mortar, one with natural aggregates and 30% fly ash as binder substitution, and another cement-based one with 100% recycled brick aggregates. Powdered brick-containing mortar was inferior in terms of fresh and hardened mortar behavior to both reference and fly ash-containing mortars, but its bond strength was significantly higher and has been shown to be surpassed only by recycled aggregate mortar [80].

Another study has investigated cement replacement in masonry mortars by up to 40% of brick waste [81]. Kumavat and Sonawane noticed a decreasing fresh and hardened mortar bulk density trend, whereas the early (up to 7 days) compressive strength of these mortars was slightly higher for mortars containing up to 15% of brick waste fines. At 28 days, it followed a declining trend for increasing the cement replacement level.

##### Cement—Masonry and Ceramic CDW—Aggregate Replacement

Aggregate substitution by recycled masonry and ceramic waste in cementitious mortars was determined as the second most populated group within the current scoping review. Relevant studies are presented in an overview in Table 4.

**Figure 6:**
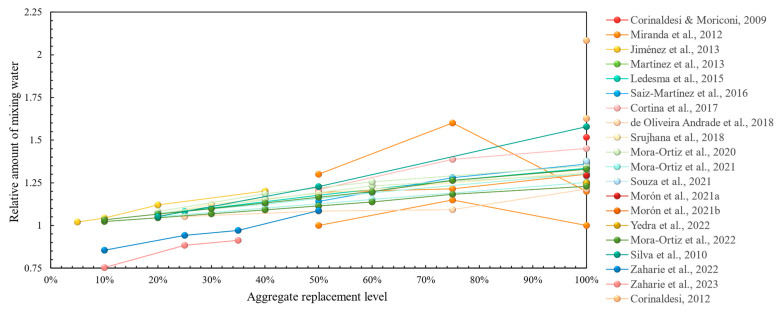
Initial mixing water requirement in recycled masonry and ceramic aggregate mortars vs. replacement levels. [47,48,53,56,59,60,62,64,82,83,85,86,88,90,91,92,93,94,95,96].

In this category, the results pointed to largely similar behavior in terms of fresh mortar properties when compared to the previous case of concrete CDW (Table 2), as the majority of recycled mortars showed a higher water demand (Figure 6) and thus, lower bulk density (Table 5). The water demand was lower only in the works of Zaharie et al. [94,95], which the authors explained by better compactness and, so a higher plasticity of mortars containing brick and masonry waste.

In the limited number of studies that evaluated these properties, water retention was found to increase while workable life decreased. For the air content, the results were inconclusive, with lower [85,87], similar [83,89], and higher [90,91,92] reported values compared with the reference mortar.

**Figure 7:**
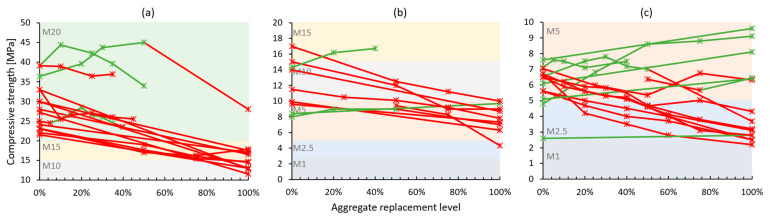
Compressive strength of recycled masonry and ceramic aggregate mortars vs. replacement levels: (**a**) M20 category reference mortars; (**b**) M15 and M10 category reference mortars; and (**c**) M1–M5 category reference mortars. Green lines and markers indicate similar or better performance compared to reference mortars, and red indicates worse performance.

Overall, the hardened properties of mortars with masonry and ceramic waste aggregates were found to follow similar trends as those incorporating recycled concrete aggregates—Table 6. Mechanical strength decreased in the majority of cases [47,48,51,53,56,59,60,64,82,85,86,90,91,92,95,96] and increased in some others [48,62,81,82,83,84,87,88,89,93,94]. Compressive strength is represented in Figure 7, and once again, the use of recycled aggregates is demonstrated to negatively affect stronger mortars (M10 strength class and above—Figure 7a,b), whereas more positive influence is attributed to them in weaker mortar formulations—Figure 7c.

Incorporation of recycled masonry and ceramic aggregates mostly decreased the density of hardened mortars, whilst increasing their shrinkage and capillary water absorption.

**Figure 8:**
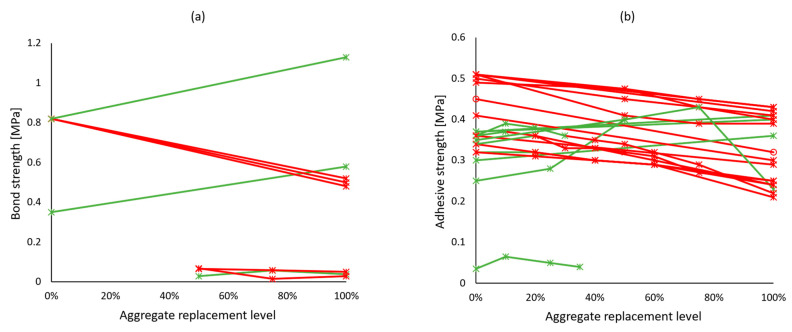
Bond strength (**a**) and adhesive strength (**b**) of recycled masonry aggregate mortars vs. replacement levels. Green lines and markers indicate similar or better performance compared to reference mortars, and red indicates worse performance.

Despite the higher overall precedence of studies considering masonry mortars as potential recipients for recycled aggregates, the most relevant masonry-scale characteristic evaluated was their adhesion—a coating mortar property. Some studies have shown encouraging results [48,62,86,92,93,95], but generally, the adhesive strength of recycled mortars was lower in comparison with the reference mortars, as demonstrated in Figure 8b [48,51,53,56,59,90,91,92,93].

Nonetheless, more studies found that the substitution of aggregates positively impacted [47,82,96] the bond strength of bricklaying mortars rather than negatively influenced it [81,95]. Rather importantly, Corinaldesi [96] proposed using a cementitious mortar with crushed red clay bricks as aggregate replacement in cultural heritage mortars, although no testing was conducted beyond a standard masonry mortar testing campaign.

##### Cement—Masonry and Ceramic CDW—Filler

In some of the earliest research on the potential utilization of recycled masonry and ceramic CDW, Miranda and Selmo [97,98] have demonstrated the key parameters for the proper use of recycled filler in rendering mortars at both mortar and masonry scales. However, this work did not present a comparable reference to better evaluate the implications of their chosen material use. Silva et al., on the other hand, have studied the performance of rendering mortars with red clay brick filler in 1:4, 1:5, and 1:6 volumetric cement to aggregate proportions (brick filler replacing 10% of natural sand) [99] and have also described another case of 1:4 renders with filler content varying from 5 to 10% [100]. In both studies, the authors have discovered improvements in fresh and hardened mortar behavior, including the adhesive strength and compatibility with substrate. Even though the water vapor permeability and shrinkage increased, renders with fine ceramic filler were declared a suitable and even superior substitution for natural aggregate mortars.

A recent attempt at comparing different fillers for masonry mortars was published by López-Uceda et al. [101]. Recycled CDW filler was based on ceramic waste, and the mix design included 500 g of CEM I type cement, 3500 g of natural aggregate, and 300 g of filler material, while superplasticizer was used to help achieve target consistency. In this study, masonry mortars with ceramic filler presented the worst performance, especially in the hardened state, compared to silicious, granite, and ornamental rock waste fillers.

#### 2.1.3. Cement—Mixed CDW

As described in the 2018 European Union waste management protocol [102], mixed CDW can include a vast array of different materials remaining after construction or generated during the demolition of structures. Such waste undergoes limited sorting, and although substantial efforts are made to separate concrete waste for reuse in new construction [103], this process can be challenging and often practically and economically unfeasible. Consequently, mixed waste remains difficult to utilize effectively [104].

##### Cement—Mixed CDW—Binder Replacement

Recycled mixed waste powder has been used as partial cement replacement of up to 30% in combination with recycled mixed waste aggregates in an experimental study by Hu et al. [105]. Authors have shown that an increase in recycled powder led to a rapid decrease in mortar performance and recommended a limited replacement value set at 20%.

##### Cement—Mixed CDW—Aggregate Replacement

The use of mixed recycled aggregates in place of natural ones in mortars for masonry has been studied alongside their concrete and masonry-based counterparts, although to a lesser extent, as demonstrated by the collection of studies in Table 7.

**Figure 9:**
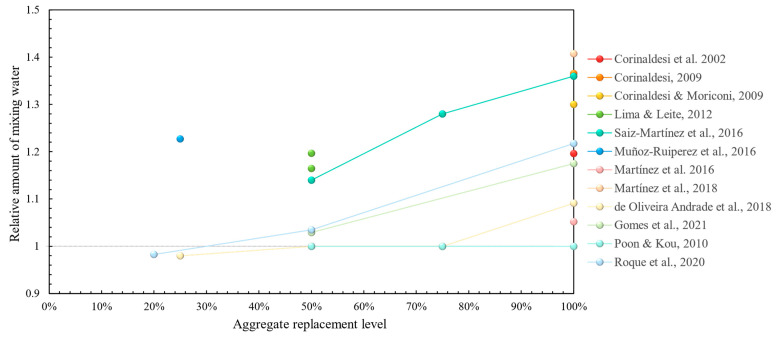
Initial mixing water requirement in recycled mixed aggregate mortars vs. replacement levels. [47,53,55,72,78,86,106,107,108,109,110,112].

Explicably, some of the works presented in previous sections included recycled mixed waste in addition to recycled concrete or masonry aggregates, which aids the explanation of extremely similar results on fresh mortar properties. In most cases, higher water demand was induced by the recycled mixed aggregates (Figure 9), although some studies reported no change compared to the reference [70,72,111,112] or even slightly lower values [86]. The reported bulk density of recycled mortars in the fresh state was lower [53,55,72,110,111], and the rest of the properties were not extensively analyzed in the present collection of research articles, as demonstrated in Table 8.

**Figure 10:**
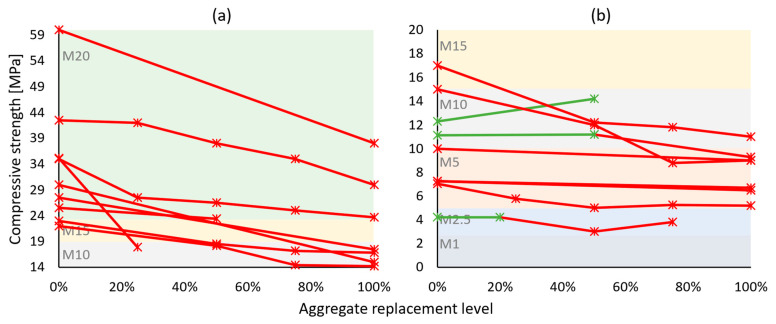
Compressive strength of recycled masonry and ceramic aggregate mortars vs. replacement levels: (**a**) M20 and M15 category reference mortars; and (**b**) M2.5–M15 category reference mortars. Green lines and markers indicate similar or better performance compared to reference mortars, and red indicates worse performance.

In terms of mechanical strength, the majority of research reported negative results, as demonstrated in Table 9 and Figure 10. This behavior was also noted for the mortars with natural aggregates substituted by recycled concrete and recycled masonry and ceramic aggregates, although in those cases, a significant portion of studies demonstrated improvement in compressive strength, especially in weaker mortars. In the case of mixed recycled aggregates, the improvement was only noted by Lima and Leite [107] and explained by the possible better interface between the recycled aggregates and the cement paste, while two more studies reported similar strength to reference samples—Figure 10b [72,110].

Few studies have shown the expected decrease in bulk density [70,86,109,110,112] and increase in shrinkage [53,107,109] and capillary water absorption [70,72,108,109,110]—Table 9.

**Figure 11:**
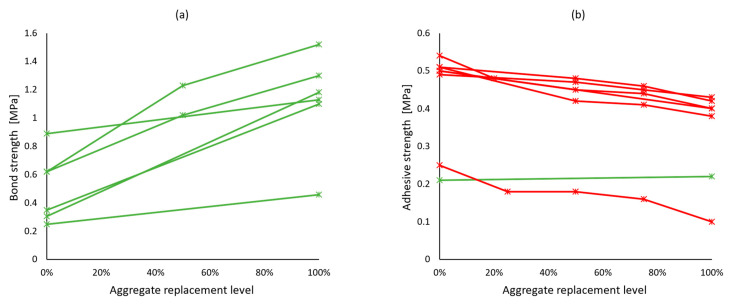
Bond strength (**a**) and adhesive strength (**b**) of recycled mixed aggregate mortars vs. replacement levels. Green lines and markers indicate similar or better performance compared to reference mortars, and red indicates worse performance.

Despite the undeniably poor mechanical performance of mortars with recycled mixed aggregates, their bond strength increased rather significantly, as shown in Figure 11a [47,70,78,79,80,106,112]. However, with mortars intended for coating purposes, the trend was largely the opposite [53,72,86,109], only with Martínez et al. [108] noticing a slight improvement in adhesive strength of mortars made with 100% of recycled mixed aggregates (Figure 11b).

These findings suggest that evaluation of recycled materials’ performance on a mortar scale (fresh and hardened properties) might be insufficient to make accurate conclusions when the intended application of mortars is in masonry construction.

##### Cement—Mixed CDW—Filler

The addition of mixed recycled CDW as an ultrafine (<125 µm) filler to masonry mortars was thoroughly studied by Ledesma et al. [113]. In fact, recycled mixed fines were used to semi and fully replace the 0–125 µm natural aggregate fraction by weight, which comprised around 5% of the total aggregate mass. The mortars were formulated using CEM II type cement in 1:4 and 1:7 cement:aggregate ratios to produce high- and low-strength bedding mortars, respectively. This replacement strategy has proven itself by overall slightly enhancing the mechanical performance of masonry mortars, while the detrimental effect on some fresh mortar properties was insignificant.

In their work on the incorporation of recycled concrete fines as fillers in cement-based rendering mortars, Jesus et al. [76,77] have also tested the addition of recycled mixed fines. The latter has shown potential in terms of fresh mortar behavior modification, such as a reduction in mixing water demand and lower fresh bulk density, suggesting a lighter mortar compared to the natural aggregate reference. With regard to hardened mortar testing of bulk density and ultrasonic pulse velocity, the performance of recycled mixed mortars was similar to that of reference mortars, whereas the dynamic modulus of elasticity was found to be increased, yet not as significantly as in the case of recycled concrete aggregates. On the other hand, mortars with mixed fine filler demonstrated inferior mechanical performance to mortars with recycled concrete filler, even though both types surpassed reference mixes. Also, a substantial decrease in capillary water absorption was observed. Unsurprisingly, larger shrinkage was noted for mortars with recycled fines, and their water permeability under pressure was higher, although the adhesive strength was better than that in reference mortars. The authors note that recycled mixed filler-containing mortars, in parallel with recycled concrete filler mortars, have demonstrated good performance and were suitable alternatives to raw silica sand fines.

#### 2.1.4. Cement—Other CDW

##### Cement—Other CDW—Binder Replacement

Even though construction and demolition waste is mostly comprised of inert mineral waste, there are integral building components that could be considered separately, i.e., insulation materials. A special case of using expanded polystyrene (EPS) waste as full cement replacement in masonry mortars has been explored by Milling et al. [114]. This experimental study presented an approach to dissolve EPS beads using acetone, effectively creating a slurry, which, when combined with sand, produced an alternative mortar. Compared to cement-based masonry mortar, this EPS-based novelty achieved lower compressive and flexural strength values, which were nevertheless considered suitable for general-purpose masonry applications. More interestingly, these EPS mortars have also demonstrated vastly superior bond strength and upon failure by compressive and tensile loading, exhibited a ductile behavior—an uncharacteristic feature of cementitious mortars.

##### Cement—Other CDW—Aggregate Replacement

The application of crushed sanitary ware as aggregates in coating mortars has been investigated by Lucas et al. [115], and while such material waste might originate from the production or use of ceramic plumbing fixtures, it could also be generated during the construction and demolition phases of buildings, albeit in relatively low amounts. The authors have explored up to 100% replacement of natural sand by crushed sanitary ware aggregates in Portland limestone cement mortars with a 1:4 volumetric binder to aggregate ratio. Experimental results of this study revealed an overall similar behavior observed for mortars with 20% and 50% of natural aggregate replacement compared to the reference one. Furthermore, 50% sanitary ware aggregate replacement in mortars increased adhesive strength despite increased shrinkage and water permeability under pressure.

Piña Ramírez et al. [116] have published a study on an innovative use of recycled mineral wool waste to partially replace sand in fire-resistant Portland limestone cement coating mortars. Recycled mineral wool fibers were obtained from different sources (Rockwool, fiberglass, and mixed) and crushed until suitable size for aggregate replacement at ~500–1000 µm. In the preparation of mortars, fibers were first added to water, then intermixed with cement, and finally, natural aggregates. In all cases, mortars were formulated using a 1:3:0.6 cement:aggregate:water ratio, with recycled mortars containing 50% fibers instead of aggregates. Mortar properties were tested before and after 1 h of open flame wood fire exposure. Mortars containing recycled fibers did not differ significantly from the reference mortars in terms of surface hardness before or after the fire exposure. On the contrary, flexural strengths were improved for rockwool and mixed fiber compositions, and more importantly, even after the fire, the flexural strength of fiber mortars was substantial, up to 10 times higher than that of the reference mortar. Compressive strength decreased compared to the reference, both before and after fire. However, the thermal conductivity of mortars with fiber incorporation was significantly lower, and interestingly, after fire exposure, it either dropped or remained the same. The authors have concluded that natural sand substitution for recycled mineral wool fiber is a viable way of increasing both the fire resistance of coating mortars and incorporating large amounts of recycled mineral wool waste in new materials.

##### Cement—Other CDW—Filler

Oliveira et al. [117] have researched the partial replacement of aggregates in rendering mortars by fine (<149 µm) waste glass. The reference mortars were made in 1:4, 1:5, and 1:6 volumetric binder to aggregate ratios, whereas recycled renderings contained 20% of glass fines relative to the total aggregate content. For all mix designs, incorporating recycled glass fines reduced water demand but increased the air content, yet led to a densification of the hardened mortar matrix. Mechanical strength was significantly increased by the 20% incorporation of fine glass, whereas the capillary water absorption decreased—both positive effects. Furthermore, the water retention and the adhesive strength of mortars with recycled glass fines also increased. The only downside to such a replacement strategy was significantly higher shrinkage in mortars with recycled glass filler.

Contributing to the investigation on the use of crushed sanitary ware, Farinha et al. have tried incorporating the finest fraction (<150 µm) of it into a cement-based rendering mortar [118]. Three different binders to aggregate volumetric ratios—1:4, 1:5, and 1:6—were studied, and for each formulation, 20% of aggregates (by volume) were replaced by sanitary ware filler. The fresh properties were improved and, rather significantly, a large increase in both compressive and flexural strengths in all mortar formulations was noted. Contrary to the results from many other studies, the dimensional instability behavior of recycled waste mortars was superior to that of reference ones, showing lower shrinkage, and so was the capillary water absorption and adhesive strength. Overall, the mortars with recycled sanitary ware fines have shown excellent performance, comparable at a higher binder to aggregate ratio to the reference 1:4 binder:aggregate mix without any recycled fines.

### 2.2. Lime-Based Binder

In this section, the main attention has been placed on the binder systems comprised of or containing lime, either hydraulic or non-hydraulic, in addition to other binding materials, such as cement, pozzolans, etc. Ascribable to the long-standing reputation of lime as a construction material [119], its application in cultural heritage and repair mortars is predictable and witnessed in many of the studies reviewed here, although envisaged applications in new construction are also prevalent in this research domain. Potentially pertaining to the prevailing use of lime in masonry construction, recycled concrete waste was not investigated as raw materials’ replacement in lime-based mortars.

#### 2.2.1. Lime—Masonry and Ceramic CDW

##### Lime—Masonry and Ceramic CDW—Binder Replacement

Lime binder substitution by recycled brick dust for the production of restoration mortars was also performed recently [120]. Interestingly, the brick dust was collected from a historic theatre and was incorporated as 10–30% hydrated lime binder replacement in restoration mortars with a 1:3 mass ratio of binder to aggregate. In this replacement scenario, the water demand decreased, and mechanical strength increased, especially at a hydrated lime replacement level of 15%. The porosity of these mortars was not significantly affected, nor was the capillary water absorption, although carbonation speed increased. Overall, the performance of lime-brick dust mortars was found acceptable for repair and restoration work.

##### Lime—Masonry and Ceramic CDW—Aggregate Replacement

Lime-based mortars have been studied less extensively when paired with masonry and ceramic CDW aggregates, compared to their cement-based counterparts. In this category, though, the split among masonry, coating, and cultural heritage mortars was almost equal, as presented in Table 10.

**Figure 12:**
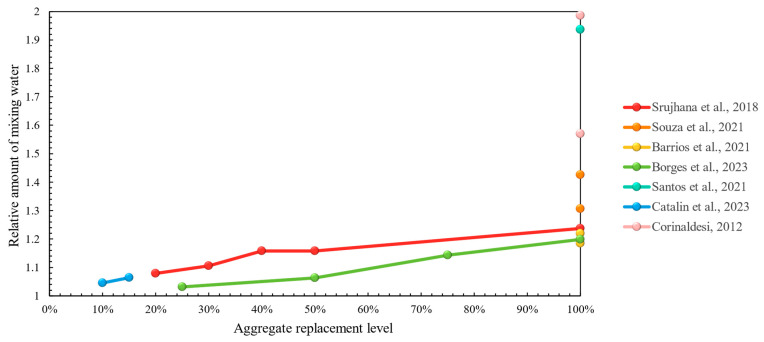
Initial mixing water requirement in lime-based recycled masonry and ceramic aggregate mortars vs. replacement levels. [62,88,96,121,122,124,125].

The limited number of studies presented limitations regarding mortar performance comparison, partly owing to the scarce results, especially for the fresh mortar state (Table 11). Based on these, only the relative amount of mixing water required to achieve targeted consistency of recycled mortars was unanimously shown to be higher than reference mortars, as demonstrated in Figure 12 [62,88,96,121,122,124,125]. Findings related to other fresh properties did not offer a basis for trend identification.

**Figure 13:**
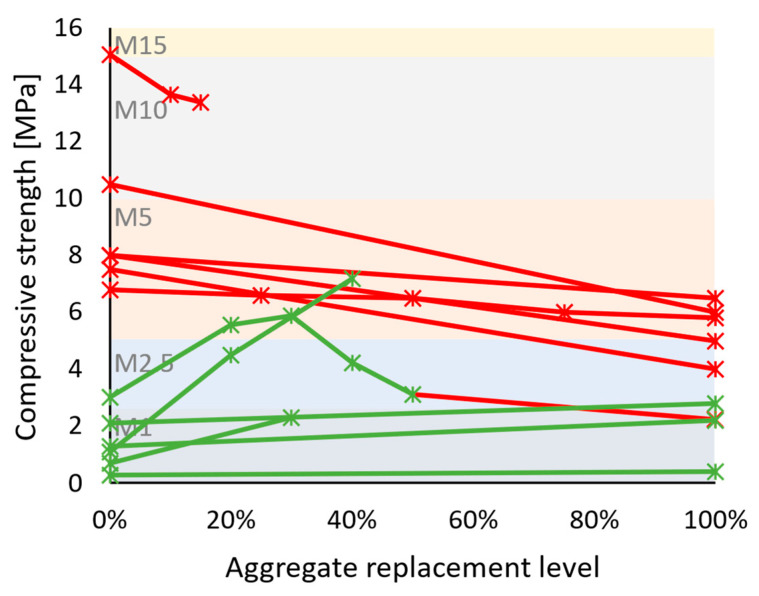
Compressive strength of lime-based recycled masonry and ceramic aggregate mortars vs. replacement levels. Green lines and markers indicate similar or better performance compared to reference mortars, and red indicates worse performance.

The properties of hardened mortar composed of lime, recycled masonry, and ceramic aggregate followed similar trends in general mortar behavior as described for cement-based mixes (Table 12). Mechanical strength was usually lower [88,96,121,122,125], yet other studies have shown improvements [62,88,124,127], as shown in Figure 13. Noticeably, the weaker lime-based mortars (M1 and M2.5 strength class) benefited from the addition of recycled masonry and ceramic CDW, which could not be attributed to the type of lime or lime and cement combination used but rather the formulation itself, once again indicating that the role of recycled aggregates could be more valuable in mixes where aggregate fraction has a relatively high importance for the strength development.

Similarly to previously presented cases, the addition of recycled masonry and ceramic aggregates decreased the bulk density of lime-based mortars [62,121,122,124,127], while capillary water absorption increased [62,96,121,122,127].

**Figure 14:**
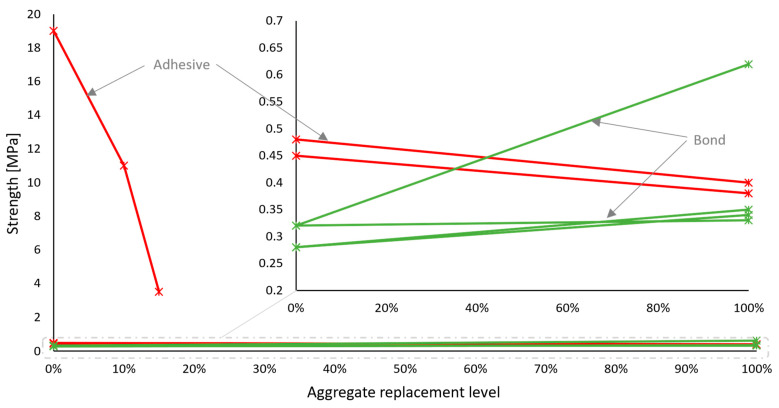
Bond strength and adhesive strength of lime-based recycled aggregate mortars vs. replacement levels. Green lines and markers indicate similar or better performance compared to reference mortars, and red indicates worse performance. The inset shows the poorly visible area of the graph.

Masonry-scale properties were not thoroughly evaluated, as shown in Figure 14, with one study suggesting improvement of bond strength [96] of a restoration mortar and two studies showing inferior adhesive strength [121,125]. However, the adhesive strength value range and rapid decline with limited natural aggregate substitution presented in [125] were not found in any previous research works.

##### Lime—Masonry and Ceramic CDW—Filler

Pozzolanic properties of brick dust were explored in a recent study on the compatibility of lime-based restoration mortars with old masonry [128]. In the study, restoration mortars were prepared using volumetric proportions of hydrated lime, brick dust, and standard sand of 1:0:2 (reference), 1:0.25:2, and 1:1:2. To compare the compatibility of these mortars with the old substrate, the authors studied dewatering behavior and water transfer by sorption, which was increased owing to the addition of brick dust, indicating potentially better bond formation between an old substrate and a new restoration masonry mortar, render, or plaster.

Another study discussed in the previous section has also presented results of utilizing recycled brick waste as filler in coating mortars [124]. The mortars were made in a 1:2:1 volumetric ratio of CL90-S hydrated lime, siliceous aggregate, and recycled brick dust, respectively. Compared to the reference, recycled mortars required a similar mixing water amount, but produced a denser fresh and hardened mortar structure. Their elastic modulus, flexural, and compressive strengths were also significantly higher than those of a reference mortar. These results have highlighted the potential application of brick dust in low-strength coating mortars.

#### 2.2.2. Lime—Mixed CDW

##### Lime—Mixed CDW—Aggregate Replacement

Mixed construction and demolition waste used as an aggregate fraction in lime-based mortars for masonry is the least populated expanded category of this review. Relevant studies are presented in Table 13.

**Figure 15:**
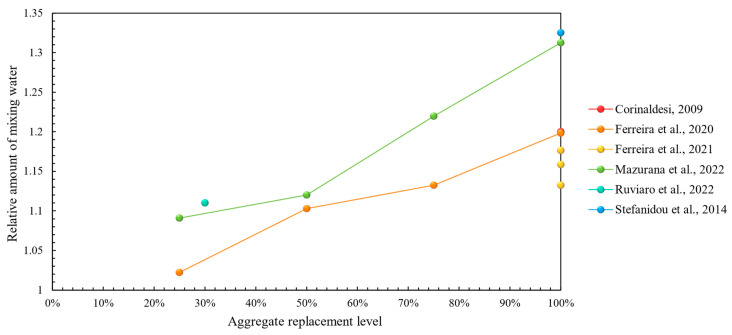
Initial mixing water requirement in lime-based recycled mixed aggregate mortars vs. replacement levels [106,129,131,132,133,134].

Based on the reported results of fresh mortar properties, all studies of this section have demonstrated higher mixing water requirement for recycled mortars, as illustrated in Figure 15, whereas other properties were not studied considerably—Table 14.

**Figure 16:**
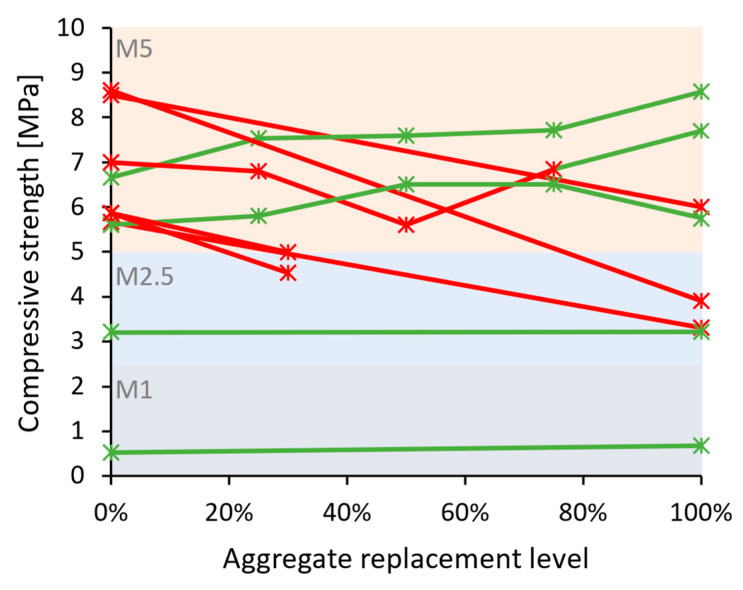
Compressive strength of lime-based recycled mixed aggregate mortars vs. replacement levels. Green lines and markers indicate similar or better performance compared to reference mortars, and red indicates worse performance.

Hardened mortar properties have presented a familiar situation, where mechanical properties were both impaired [106,130,131,133] and improved [129,130,132,134] by the addition of recycled mixed aggregates—Table 15. However, this type of recycled material has been demonstrated to have had largely negative impacts on cement-based mortars (Section 2.1.3). In weaker lime-based mixtures (Figure 16), the positive contributions were equally matched with the negative effects that concerned the mechanical properties of the mortar. In addition, recycled mortars could be characterized as having a lower density [129,132,133], yet shrinkage was higher according to one study [133], and in terms of capillary water absorption, no trend could be established.

**Figure 17:**
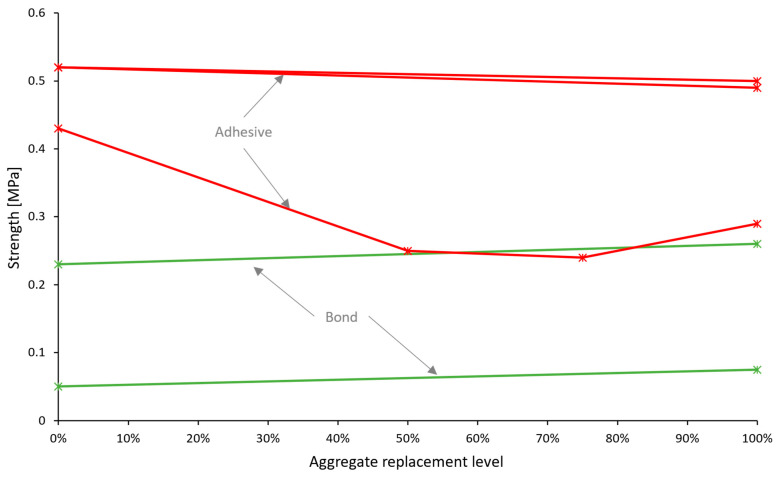
Bond strength and adhesive strength of lime-based mixed recycled aggregate mortars vs. replacement levels. Green lines and markers indicate similar or better performance compared to reference mortars, and red indicates worse performance.

Similarly to all the previous cases, the addition of mixed aggregates to lime-based mortars resulted in an improved bond strength [106,134] and decreased [130] or very similar [133] adhesive strength (Figure 17).

#### 2.2.3. Lime—Other CDW

##### Lime—Other CDW—Binder Replacement

As part of their investigation into hydrated lime replacement for restoration purposes, Ayat et al. [120] have also explored the addition of waste glass powder, similarly as described previously for the case of brick dust. The performance of mortars with glass powder was similar to the performance of brick dust-containing hydrated lime mortars, allowing for the same conclusions regarding the suitability of mortar for the intended renovation purposes.

##### Lime—Other CDW—Filler

Thermal insulation waste has been explored as filler for restoration and coating mortars made with lime binders [135]. These mortars were designed using hydrated lime to aggregate a mass proportion of 1:3, with additions of different EPS beads and recycled mineral wool at roughly 0.55% and 1% relative to the binder amount. These small additions of thermal insulation waste induced higher water demand for the right mortar consistency. In terms of mechanical performance, mortars with both EPS types have demonstrated a decrease, whereas increased flexural and compressive strength was measured in mortars with recycled mineral wool additions. However, the adhesive strength was reduced significantly in comparison with the reference mortar. On a positive note, shrinkage was minimized, whilst thermal properties were enhanced.

### 2.3. Other Binders

#### 2.3.1. Other—Concrete CDW

##### Other—Concrete CDW—Binder Replacement

The application of recycled concrete powder to replace limestone during masonry cement production was proposed as a simultaneous natural resource use minimizing and waste material valorization measure [136]. This new, recycled concrete aggregate-containing masonry cement was then assessed based on masonry mortar performance. This study has shown that when ground to similar fineness, both natural limestone and recycled concrete powder can be used interchangeably in masonry cement production.

##### Other—Concrete CDW—Aggregate Replacement

Masonry mortars, based on alkali-activated materials, were studied in conjunction with the use of recycled concrete aggregates for their potential for carbon dioxide sequestration [137] and for possible performance-based substitution [138]. In the first case, authors have concluded that the utilization of waste aggregates was essential for the effective sequestration of atmospheric CO_2_ by these newly developed mortars. In the other study [138], metakaolin–limestone binders activated using sodium hydroxide and sodium silicate were used in mortars with recycled aggregates, which increased the activator demand and water retention, while their mechanical strength suffered a decrease, and so did the adhesive strength when replacement levels exceeded 20%.

In their study on plastering mortars with recycled concrete aggregates, Ferrández et al. [139] used commercial gypsum plaster supplemented by natural or recycled aggregates. The performance of such mortars with recycled aggregates diminished considerably, except for the thermal properties.

#### 2.3.2. Other—Masonry and Ceramic CDW

##### Other—Masonry and Ceramic CDW—Binder Replacement

A more recent study has showcased the potential of pulverized clay brick and mortar to be used in alkali-activated masonry mortars [140]. This system contained crushed bricks, natural aggregates, and alkaline activators (sodium hydroxide and sodium silicate in varying proportions). A similar mix with fly ash instead of brick powder was evaluated for comparison between industrial waste and CDW. The results of this study revealed that crushed brick masonry mortars exhibited worse performance than fly ash-based mortars. Another study on alkali-activated mortars, comprised of various combinations of lime, ground granulated blast furnace slag, and brick powder, paired with natural aggregates in a 1:2 mass ratio, has demonstrated great performance of such materials, with a very strong focus on the masonry-scale properties of mortars designed for restoration purposes [141].

##### Other—Masonry and Ceramic CDW—Aggregate Replacement

Ferrández et al. [139] have also investigated natural sand replacement by recycled masonry aggregates in commercial gypsum plasterormulations. In terms of mortar properties, the use of recycled masonry aggregates negatively impacted most, with subpar experience compared to reference and recycled concrete aggregate mixes. Only the thermal performance was enhanced even further than with recycled concrete. On the other hand, commercial gypsum plasters, supplemented by recycled ceramics as coarse and fine aggregates, exhibited superior performance to the plastering mixes without aggregates [142]. Even though the effect of adding crushed red clay brick could not be measured against natural aggregate addition, the authors have shown that using aggregates in ready-to-use plastering mortars can result in significant improvement in their performance, including important properties, such as adhesion.

Other recent attempts at producing sustainable plastering mortars have been documented by Xu et al. [143]. Recycled waste clay bricks originating from demolished buildings have been crushed and used as a partial replacement for natural aggregates in plastering mortars. These mortars were prepared using Portland cement–fly ash binder combinations in a 1:4 binder to aggregate ratio by mass. All mortars also contained hydroxypropyl methyl cellulose as a thickening and water retention-increasing additive. Despite the higher shrinkage in recycled aggregate mortars, their strength was higher or on par with that of reference, and similar results were reported for the tensile bond strength.

### 2.4. Environmental Performance

Even though the topic of waste material utilization revolves around the idea of it being sustainable, most studies reviewed here simply assume that “recycled” equals “more sustainable than virgin materials”. However, in the case of recycled aggregates, the environmental benefits associated with their use could be doubtful, depending on particular circumstances [144,145].

In this review, only a few works have considered the life cycle assessment of proposed mortar formulations. Cortina et al. [56] and Cuenca-Moyano et al. [146,147] revealed the possibilities of achieving environmental benefits through the incorporation of recycled concrete and recycled masonry and ceramic aggregates in cement-based masonry mortars.

Recycled ceramic aggregate production has a significantly lower carbon footprint than natural aggregate production, according to findings from Cortina et al. [56]. The authors calculated a reduction of 13.5 times in terms of CO_2_-equivalent emissions produced per kg of recycled aggregates, compared to a generic value from the environmental impact assessment database for natural aggregates. Even though no further calculations relating to mortars or masonry built using these aggregates were made, the obtained result was claimed to prove the valuable contribution of recycled aggregates towards sustainable developments in the built environment.

A more holistic approach to environmental performance assessment for masonry mortars was developed [146] and demonstrated [147] by Cuenca-Moyano et al. Firstly, the authors have demonstrated that all the environmental burdens across various impact categories [148] arising from the production of recycled fine concrete aggregates can be balanced out considering positive contributions from the avoided landfilling and transportation of such materials, presenting possibilities to achieve environmental gains or net-negative impacts. Furthermore, when such aggregates were utilized to replace 25% of the natural aggregate mass in masonry mortars (1:9 cement:aggregate mass ratio, strength class M5), their environmental impacts were found to be lower in most categories when compared to natural aggregate mortars, thus providing a clear proof of environmentally feasible application of recycled fine concrete aggregates in new masonry mortars, under the particular scenarios considered in this work [147].

## 3. Conclusions and Research Implications

The variability of previously researched applications of recycled building materials in mortars for masonry demonstrates the multi-faceted nature of this pressing topic. After a comprehensive overview and structuring of the state-of-the-art contributions, some specific areas presenting enough data for systematic analysis and higher-certainty conclusions were identified. Contrastingly, many of the discussed application cases for particular raw and recycled materials and their incorporation types in mortars were studied insufficiently to reach reliable inferences. The following sections discuss the positive aspects discovered in this scoping review, as well as the identified limitations, along with the opportunities and challenges they present in a broader context.

### 3.1. Positive Aspects

Cement-based masonry mortars formulated with recycled concrete, recycled masonry and ceramic, and recycled mixed aggregates present the most researched groups within the domain of interest. Considered jointly or separated into each type of recycled material, these studies could present a well-rounded basis for a specific systematic data analysis, allowing scientists, industry practitioners, and legislators to develop a better understanding of recycled materials and their effects on masonry mortars.

Simple linear regression analysis, included in Supplementary Data, has shown that in cement-based mortars for masonry, natural aggregate replacement by recycled concrete aggregates fared best in terms of mortar compressive strength when compared to recycled masonry and ceramic and recycled mixed aggregates (Tables S3, S6 and S9 and Figures S1, S3 and S5 in Supplementary Materials). This is despite the fact that recycled mixed aggregates required less additional water during mixing to achieve similar fresh behavior (Figure S6). Considering the full scope of reviewed results, summarized in the Supplementary Data file, complete aggregate replacement in cement-based mortars could predictably increase water demand by 20–34% and yield strength reduction of 11–50%, with recycled concrete aggregates being the most favorable.

Where data were plentiful to allow for the identification of trends, cement-based mortars with lower binder proportions, i.e., the mortars suitable for various masonry applications, with strengths lower than 10 MPa, have demonstrated more adequate receptibility of recycled materials, often presenting better mortar and/or masonry-scale properties.

Furthermore, lime-based mortars with natural aggregate replacement by recycled masonry and ceramic aggregates and recycled mixed aggregates were at 65% and 90% of the reference mortar strength (Figures S7 and S9), respectively, while still requiring 42% and 22% more mixing water (Figures S8 and S10). Even though reviewed data were not extensive enough for high-certainty conclusions, established trends are nevertheless important indicators of the potential effects of such recycled aggregate types in lime-based materials.

Finally, environmental performance evaluations, in spite of limited availability, have showcased the potentially achievable benefits of recycled material production and use in mortars for masonry applications.

### 3.2. Limitations

The inconsistency of results, even within the well-researched groups mentioned above, and even more so in the “niche” areas presented in this scoping review, is the main drawback of the current state of the art. Arising from geographical, social, economic, and political factors, this variability can only be addressed by the extensive and thorough interchange of information on the best local practices in the global context.

Many studies, especially those limited to material-level experiments, lack relevance in connecting to a particular application in masonry construction, which often requires specific properties that are not fully addressed at a mortar level. Furthermore, none of the studies present significant findings from outside the laboratory environment, complicating the assessment of real-world properties, such as structural performance, long-term durability, maintenance requirements, impacts on building physics, and human health.

The environmental performance assessment is omitted in most studies with a de facto assumption that recycled materials are inherently environmentally superior to raw materials. However, there are cases in which such an assumption is false, as demonstrated in one study via the uncertainty and sensitivity analysis. Overall, many factors influence the environmental evaluation of recycled materials, including their type, logistics, processing requirements, economic value, performance, and durability, all of which need to be considered case-by-case. These aspects have not been adequately addressed in reviewed studies.

### 3.3. Possibilities for Research Dissemination and Valorization

Even though the current limitations of recycled materials are highly reflected in research, this topic is a direct application of the principles of circularity in construction. Furthermore, producers increasingly seek profitable ways to use materials that typically end up in landfills, while consumers strive to adopt more environmentally conscious practices. This combined demand stands to benefit both groups. Paired with rising regulatory pressure and economic drivers, various applications of recycled building materials in the masonry industry could develop into a significant part of more sustainable construction.

### 3.4. Risk Considerations

The inherent problem of recycled materials is the quality assurance in light of heterogeneity, which has been proven to limit the scientific community’s efforts to develop a comprehensive understanding of these materials’ effects when applied in new masonry construction. More importantly, the recycling industry faces difficulties in selling or utilizing its production without such knowledge. Despite the efforts of researchers and industry professionals, suitable solutions might not be developed in due time, therefore leaving a growing problem in the rapidly developing world. Furthermore, even if solutions become readily available, their implementation at scale might face a regulatory barrier, which comes at a significant cost of time.

## Figures and Tables

**Table 1:** Summary of studies on masonry mortars and coating mortars with natural aggregate replacement by recycled concrete aggregates.

Mortar Type	Study	Binder Type	Mortar Mix	Concrete Waste Specifics and Replacement Ratio	Special Additions
MASONRY MORTAR	Vegas et al., 2009 [46]	CEM II/B-M(V-S-LL) 42.5R cement	1:10 binder to aggregate ratio by mass	Lower-quality concrete waste 10, 20, 25, 50, 75, and 100% replacement	Water retaining and superplasticizer admixtures
	Corinaldesi & Moriconi, 2009 [47]	CEM II/B-L 32.5R cement	1:3 binder to aggregate ratio by mass	Precast concrete industry waste, processed in the laboratory 100% replacement	
	Martínez et al., 2013 [48]	Portland cement P-350 (Cuba)	1:4:2 volumetric ratio of cement:aggregate:filler for natural aggregates; 1:5:1 for recycled aggregates	Concrete CDW from structural parts of demolished housing 100% replacement	Hydrated lime, Limestone, and white slag fillers
	Ledesma et al., 2014 [49]	CEM IV/A (V) 32.5N cement	1:7 binder to aggregate ratio by volume	Concrete CDW screened to 0/8 mm size 5, 10, 20, and 40% replacement	Plasticizer admixture
	Cuenca-Moyano et al., 2014 [50]	CEM II/A-L 42.5R cement	1:9 binder to aggregate by mass	Concrete CDW 25, 50, 75, and 100% replacement	Air entraining/plasticizing admixture Limestone filler
	Saiz-Martínez et al., 2015 [51]	CEM II/B-L 32.5N cement	1:3 binder to aggregate ratio by mass	Concrete CDW 10, 15, 25, 35, and 45% replacement	Superplasticizer additive
	Raeis Samiei et al., 2015 [52]	CEM II/B-P 32.5 cement	1:3 binder to aggregate ratio by mass	Laboratory-made concrete waste 25, 50, 75, and 100% replacement	
	Saiz-Martínez et al., 2016 [53]	CEM II/B-L 32.5N and CEM IV/B (V) 32.5N cement	1:3 and 1:4 binder to aggregate ratio by mass	Concrete CDW 50, 75, and 100% replacement	Superplasticizer additive
	Fernández-Ledesma et al., 2016 [54]	CEM II/B-L 32.5N cement	1:5 binder to aggregate ratio in volume	Concrete CDW screened to 0/8 mm size 2 5, 50, 75, and 100% replacement	Plasticizer admixture
	Muñoz-Ruiperez et al., 2016 [55]	CEM I 42.5R cement	1:4 binder to aggregate ratio by mass	Prefabricated concrete waste 25% (volume) replacement	Lightweight expanded clay aggregate
	Cortina et al., 2017 [56]	CEM IV/B (V) 32.5N cement	1:3 and 1:4 binder to aggregate ratio by mass	Concrete CDW 50, 75, and 100% replacement	Superplasticizer additive
	Berredjem et al., 2018 [57]	CEM I 52.5 cement	1:3 binder to aggregate ratio by mass	Lab-made concrete specimens, crushed 15, 30, 40, 50, 75, and 100% replacement	Superplasticizer additive
	Cuenca-Moyano et al., 2020 [58]	CEM II/A-L 42.5R cement	1:9 binder to aggregate by mass	Concrete CDW 25, 50, 75, and 100% replacement	Air entraining/plasticizing admixture Limestone filler
	Morón et al., 2021a [59]	CEM IV/B (P-V) 32.5N cement	1:3 binder to aggregate ratio by mass	Concrete CDW 100% replacement	Synthetic fibers Superplasticizer additive
	Morón et al., 2021b [60]	CEM II/B-L 32.5N cement	1:3 binder to aggregate ratio by mass	Concrete CDW 100% replacement	Steel fibers Superplasticizer additive
	Morón et al., 2021c [61]	CEM II/B-L 32.5N cement	1:3 and 1:4 binder to aggregate ratio by mass	Concrete CDW 100% replacement	Aramid fibers Superplasticizer additive
	Souza et al., 2021 [62]	Portland pozzolanic cement CP IV-32 (Brazil)	1:3 and 1:5 binder to aggregate ratio by volume	Structural concrete CDW 100% replacement	
	Kaplan et al., 2021 [63]	CEM I 42.5R cement	1:5, 1:6, and 1:7 binder to aggregate ratio by mass	Concrete CDW 25, 50, 75, and 100% replacement	
	Yedra et al., 2022 [64]	CEM II/B-L 32.5N cement	1:3 and 1:4 binder to aggregate ratio by mass	Concrete CDW 100% replacement	Superplasticizer additive
	Fernández et al., 2022b [65]	CEM II/B-M (V-L) 32.5N cement	1:3 binder to aggregate ratio by mass	Concrete CDW 100% replacement	Superplasticizer additive
	Murthi et al., 2022 [66]	Portland Pozzolana Cement (India)	1:6 binder to aggregate ratio by volume	Concrete CDW 10, 20, 30, and 40% replacement	
	da Magalhães & Pinto, 2023 [67]	Portland cement CPII F-32 (Brazil)	1:3 binder to aggregate ratio by mass	Lab-made hydrated cement waste 25, 50, 75, and 100% replacement	
	Galderisi et al., 2023 [68]	CEM I 42.5 R cement	1:2 binder to aggregate ratio by mass	Earthquake concrete rubble 50 and 100% replacement	
	Vaishnav & Trivedi, 2023 [69]	OPC-43 cement (India)	1:3 binder to aggregate ratio by mass	Concrete CDW, crushed and milled in the lab 25, 50, 75, and 100% replacement	Polycarboxylate ether superplasticizer
COATING MORTAR	Kou & Poon, 2013 [70]	Ordinary Portland cement	1:3 binder to aggregate ratio by mass	Concrete CDW 25, 50, 75, and 100% replacement	
	Neno et al., 2014 [71]	CEM II/B-L 32.5 N	1:4 binder to aggregate ratio by volume	Lab-made concrete C30/37, crushed 20, 50, and 100% replacement	
	Roque et al., 2020 [72]	CEM II/B-L 32.5 N	1:4 binder to aggregate ratio by volume	Concrete waste 20, 50, and 100% replacement	
	Kaplan et al., 2021 [63]	CEM I 42.5R cement	1:5, 1:6, and 1:7 binder to aggregate ratio by mass	Concrete CDW 25, 50, 75, and 100% replacement	
	Garg & Shrivastava, 2023 [73]	Cement	1:3 binder to aggregate ratio by volume	Concrete CDW 25, 50, 75, and 100% replacement	

**Table 2:** Performance of recycled concrete aggregate mortars based on fresh mortar properties, as reported in reviewed studies.

Comparison with Reference Mortars with Natural Aggregates	Fresh Mortar Properties				
	Water Requirement for Similar Consistency	Bulk Density	Air Content	Water Retention	Workable Life
Lower value	0	16	1	0	3
Similar value	2	0	4	0	0
Higher value	26	2	2	4	1

**Table 3:** Performance of recycled concrete aggregate mortars based on hardened mortar properties, as reported in reviewed studies.

Comparison with Reference Mortars with Natural Aggregates	Hardened Mortar Properties							
	Flexural Strength	Compressive Strength	Bulk Density	Shrinkage	Capillary Water Absorption	Bond Strength	Adhesive Strength	Water Vapor Permeability
Lower value	18	21	16	1	3	0	5	1
Similar value	1	1	1	2	2	0	0	1
Higher value	10	10	5	13	12	3	3	3

**Table 4:** Summary of studies on cement-based masonry, coating, and cultural heritage mortars with natural aggregate replacement by recycled masonry and ceramic aggregates.

	Study	Binder Type	Mortar Mix	Masonry and Ceramic Waste Specifics and Replacement Ratio	Special Additions
MASONRY MORTAR	Corinaldesi & Moriconi, 2009 [47]	CEM II/B-L 32.5R cement	1:3 binder to aggregate ratio by mass	New red bricks, crushed and processed in the laboratory 100% replacement	
	Miranda et al., 2012 [82]	Portland-pozzolana cement (Brazil)	1:8.5, 1:8.25, and 1:8 binder to aggregate ratio by mass	Masonry waste from construction sites, processed in the lab to 1.2 mm max particle size 50, 75, and 100% replacement	
	Jiménez et al., 2013 [83]	CEM IV/A (V) 32.5N cement	1:7 binder to aggregate ratio by volume	Ceramic partition wall waste 5, 10, 20, and 40% replacement	Air entraining/plasticizing admixture
	Martínez et al., 2013 [48]	Portland cement P-350 (Cuba)	1:4:2 volumetric ratio of cement:aggregate:filler for natural aggregates; 1:5:1 for recycled aggregates	Mortar and ceramic CDW 100% replacement	Hydrated lime, Limestone, and white slag fillers
	Kumavat & Sonawane, 2013 [81]	Cement	1:4 binder to aggregate ratio by mass	Brick waste 5, 10, 15, 20, 25, 30, 35, and 40% replacement	
	Nie et al., 2014 [84]	Ordinary Portland cement (China)	Unknown binder to aggregate ratio	Ceramic tile waste 25, 50, 75, and 100% replacement	
	Ledesma et al., 2015 [85]	CEM II/B-L 32.5N cement	1:5 binder to aggregate ratio in volume	Masonry CDW 25, 50, 75, and 100% replacement	Plasticizer admixture
	Saiz-Martínez et al., 2015 [51]	CEM II/B-L 32.5N cement	1:3 binder to aggregate ratio by mass	Ceramic CDW 10, 15, 25, 35, and 45% replacement	Superplasticizer additive
	Saiz-Martínez et al., 2016 [53]	CEM II/B-L 32.5N and CEM IV/B (V) 32.5N cement	1:3 and 1:4 binder to aggregate ratio by mass	Ceramic CDW 50, 75, and 100% replacement	Superplasticizer additive
	Cortina et al., 2017 [56]	CEM IV/B (V) 32.5N cement	1:3 and 1:4 binder to aggregate ratio by mass	Ceramic CDW 50, 75, and 100% replacement	Superplasticizer additive
	de Oliveira Andrade et al., 2018 [86]	Portland pozzolanic cement (Brazil)	1:4 binder to aggregate ratio by volume	Ceramic CDW 25, 50, 75, and 100% replacement	
	Silva Neto & Leite (2018) [87]	CPV-ARI cement (Brazil)	1:5 binder to aggregate ratio by mass	Rendering mortar waste 20 and 40% replacement	
	Srujhana et al., 2018 [88]	Cement	1:6 binder to aggregate ratio by mass	Demolished masonry waste 20, 30, 50, 50, and 100% replacement	
	Evangelista et al., 2019 [89]	CPV-ARI cement (Brazil)	1:3 binder to aggregate ratio by mass	Ceramic CDW 20, 30, 50, and 100% replacement	
	Mora-Ortiz et al., 2020 [90]	PCC 30R type cement (Mexico)	1:4 binder to aggregate ratio by volume	Demolition masonry waste 20, 40, 60, and 100% replacement	Plasticizer admixture and aggregate pre-wetting
	Mora-Ortiz et al., 2021 [91]	PCC 30R type cement (Mexico)	1:4 binder to aggregate ratio by mass	Demolition masonry waste 20, 40, 60, and 100% replacement	Aggregate pre-wetting
	Souza et al., 2021 [62]	Portland pozzolanic cement CP IV-32 (Brazil)	1:3 and 1:5 binder to aggregate ratio by volume	Masonry CDW 100% replacement	
	Morón et al., 2021a [59]	CEM IV/B (P-V) 32.5N cement	1:3 binder to aggregate ratio by mass	Ceramic CDW 100% replacement	Synthetic fibers Water reducing and set accelerating admixture
	Morón et al., 2021b [60]	CEM II/B-L 32.5N cement	1:3 binder to aggregate ratio by mass	Ceramic CDW 100% replacement	Steel fibers Superplasticizer additive
	Yedra et al., 2022 [64]	CEM II/B-L 32.5N cement	1:3 and 1:4 binder to aggregate ratio by mass	Ceramic CDW 100% replacement	Superplasticizer additive
	Mora-Ortiz et al., 2022 [92]	PCC 30R type cement (Mexico)	1:4 binder to aggregate ratio by volume	Mortar and red clay brick waste 10, 20, 30, 40, 50, 60, 80, and 100% replacement	Aggregate pre-wetting
COATING MORTAR	Silva et al., 2010 [93]	Cement	1:4 binder to aggregate ratio by volume	Red clay brick waste 20, 50, and 100% replacement	
	Zaharie et al., 2022 [94]	CEM I 52.5R cement	1:4 binder to aggregate ratio by mass	Red clay brick waste 10, 25, 35, and 50% replacement	
	Zaharie et al., 2023 [95]	CEM I 52.5R cement	1:4 binder to aggregate ratio by mass	Masonry waste 10, 25, and 35% replacement	
CULTURAL HERITAGE	Corinaldesi, 2012 [96]	CEM II/B-L 32.5R cement	1:3 binder to aggregate ratio by mass	New red bricks, crushed and processed in the laboratory 100% replacement	

**Table 5:** Performance of recycled masonry and ceramic aggregate mortars based on fresh mortar properties, as reported in reviewed studies.

Comparison with Reference Mortars with Natural Aggregates	Fresh Mortar Properties				
	Water Requirement for Similar Consistency	Bulk Density	Air Content	Water Retention	Workable Life
Lower value	2	14	2	0	3
Similar value	0	0	2	1	0
Higher value	22	1	3	3	0

**Table 6:** Performance of recycled masonry and ceramic aggregate mortars based on hardened mortar properties, as reported in reviewed studies.

Comparison with Reference Mortars with Natural Aggregates	Hardened Mortar Properties							
	Flexural Strength	Compressive Strength	Bulk Density	Shrinkage	Capillary Water Absorption	Bond Strength	Adhesive Strength	Water Vapor Permeability
Lower value	13	18	17	1	3	2	9	1
Similar value	2	2	0	0	0	0	1	0
Higher value	9	10	2	7	12	3	6	4

**Table 7:** Summary of studies on masonry and coating mortars with natural aggregate replacement by recycled mixed aggregates.

	Study	Binder Type	Mortar Mix	Masonry and Ceramic Waste Specifics and Replacement Ratio	Special Additions
MASONRY MORTAR	Corinaldesi et al., 2002 [78]	CEM II/A-L 42.5R cement	1:3 binder to aggregate ratio by mass	Mixed waste 100% replacement	Stainless steel and polypropylene fibers
	Moriconi et al., 2003 [79]	CEM II/A-L 42.5R cement	1:3 binder to aggregate ratio by mass	Mixed waste 100% replacement	
	Corinaldesi & Moriconi, 2004 [80]	CEM II/A-L 42.5R cement	1:3 binder to aggregate ratio by mass	Mixed waste 100% replacement	
	Corinaldesi, 2009 [106]	CEM II/A-L 3.5R cement	1:3 binder to aggregate ratio by mass	Mixed waste 100% replacement	
	Corinaldesi & Moriconi, 2009 [47]	CEM II/B-L 32.5R cement	1:3 binder to aggregate ratio by mass	Mixed waste 100% replacement	
	Lima & Leite, 2012 [107]	Portland cement CP V ARI (Brazil)	1:4 and 1:8 binder to aggregate ratio by mass	Mixed CDW 50% replacement	
	Saiz-Martínez et al., 2016 [53]	CEM II/B-L 32.5N and CEM IV/B (V) 32.5N cement	1:3 and 1:4 binder to aggregate ratio by mass	Mixed CDW 50, 75, and 100% replacement	Superplasticizer additive
	Muñoz-Ruiperez et al., 2016 [55]	CEM I 42.5R cement	1:4 binder to aggregate ratio by mass	Mixed CDW 25% (volume) replacement	Lightweight expanded clay aggregate
	Martínez et al., 2016 [108]	Portland cement P-350 (Cuba)	1:4:2 volumetric ratio of cement:aggregate:filler for natural aggregates; 1:5:1 for recycled aggregates	Mixed CDW 100% replacement	Limestone filler
	Martínez et al., 2018 [109]	Portland cement P-350 (Cuba)	1:4:2 volumetric ratio of cement:aggregate:filler for natural aggregates; 1:5:1 for recycled aggregates	Mixed CDW 100% replacement	Hydrated lime and limestone filler
	de Oliveira Andrade et al., 2018 [86]	Portland pozzolanic cement (Brazil)	1:4 binder to aggregate ratio by volume	Mixed CDW 25, 50, 75, and 100% replacement	
	Gomes et al., 2021 [110]	CEM II/B-L 32.5N cement	1:4 binder to aggregate ratio by volume	Mixed CDW (commercialized in Portugal) 50 and 100% replacement	
	Cangussu et al., 2022 [111]	CP IV 32 cement (Brazil)	~1:5.9:1.4 binder to aggregate to water mass ratio	Mixed CDW 100% replacement	
COATING MORTAR	Poon & Kou, 2010 [112]	Ordinary Portland cement	1:3 binder to aggregate ratio by mass	Mixed CDW 25, 50, 75, and 100% replacement	
	Kou & Poon, 2013 [70]	Ordinary Portland cement	1:3 binder to aggregate ratio by mass	Concrete and brick CDW 25, 50, 75, and 100% replacement	
	Roque et al., 2020 [72]	CEM II/B-L 32.5 N	1:4 binder to aggregate ratio by volume	Mixed waste 20, 50, and 100% replacement	

**Table 8:** Performance of recycled mixed aggregate mortars based on fresh mortar properties, as reported in reviewed studies.

Comparison with Reference Mortars with Natural Aggregates	Fresh Mortar Properties				
	Water Requirement for Similar Consistency	Bulk Density	Air Content	Water Retention	Workable Life
Lower value	1	5	0	0	0
Similar value	4	0	0	2	0
Higher value	11	0	1	1	0

**Table 9:** Performance of recycled mixed aggregate mortars based on hardened mortar properties, as reported in reviewed studies.

Comparison with Reference Mortars with Natural Aggregates	Hardened Mortar Properties							
	Flexural Strength	Compressive Strength	Bulk Density	Shrinkage	Capillary Water Absorption	Bond Strength	Adhesive Strength	Water Vapor Permeability
Lower value	13	14	5	1	0	0	4	0
Similar value	1	2	0	1	1	0	0	0
Higher value	0	1	0	3	5	5	1	1

**Table 10:** Summary of studies on lime-based masonry, coating, and cultural heritage mortars with natural aggregate replacement by recycled masonry and ceramic aggregates.

	Study	Binder Type	Mortar Mix	Masonry and Ceramic Waste Specifics and Replacement Ratio	Special Additions
MASONRY MORTAR	Srujhana et al., 2018 [88]	Cement and lime	1:1:12 cement to lime to aggregate ratio by mass	Demolished masonry waste 20, 30, 50, 50, and 100% replacement	
	Souza et al., 2021 [62]	Portland pozzolanic cement CP IV-32 and hydrated lime CH III (Brazil)	1:2:6 and 1:2:8 cement to lime to aggregate ratio by volume	Masonry CDW 100% replacement	
	Barrios et al., 2021 [121]	CEM II/B-L 32.5N cement and CL70 hydrated lime	1:1:6 and 1:1:8 cement:lime:aggregate ratio by mass	Recycled ceramic aggregates 100% replacement	Fibers, Superplasticizer admixture
	Borges et al., 2023 [122]	Portland cement CPV-ARI and hydrated lime CH III (Brazil)	1:1:6 cement:lime:aggregate ratio by mass	Ceramic waste 25, 50, 75, and 100% replacement	
COATING MORTAR	Falchi et al., 2017 [123]	Natural hydraulic lime NHL3.5	1 part binder, 2 parts natural sand, 1 part recycled sand	Old mortar waste 33% replacement	Air entraining admixture
	Santos et al., 2021 [124]	Air lime CL90-S	1:3 volumetric binder to aggregate ratio for natural aggregates, 1:2 for recycled	Brick waste 100% aggregate replacement	
	Catalin et al., 2023 [125]	CEM II/A-S 52.5R cement and hydrated lime	2.5:1:13.2 cement to lime to aggregate ratio by mass	Plaster waste 10 and 15% replacement	
CULTURAL HERITAGE	Corinaldesi, 2012 [96]	Hydraulic lime	1:3 binder to aggregate ratio by mass	New red bricks, crushed and processed in the laboratory 100% replacement	
	Xu et al., 2014 [126]	Natural hydraulic lime NHL2	1:2 binder to aggregate ratio by mass	Masonry waste aggregates	Diatomite replacing masonry waste aggregates
	Torres et al., 2020 [127]	Natural hydraulic lime NHL3.5	1:3 and 1:4 binder to aggregate ratio by volume	Ceramic waste 20, 30, and 40% replacement	

**Table 11:** Performance of lime-based recycled masonry and ceramic aggregate mortars based on fresh mortar properties, as reported in reviewed studies.

Comparison with Reference Mortars with Natural Aggregates	Fresh Mortar Properties				
	Water Requirement for Similar Consistency	Bulk Density	Air Content	Water Retention	Workable Life
Lower value	0	2	0	0	0
Similar value	0	1	0	0	0
Higher value	7	0	0	1	0

**Table 12:** Performance of lime-based recycled masonry and ceramic aggregate mortars based on hardened mortar properties, as reported in reviewed studies.

Comparison with Reference Mortars with Natural Aggregates	Hardened Mortar Properties							
	Flexural Strength	Compressive Strength	Bulk Density	Shrinkage	Capillary Water Absorption	Bond Strength	Adhesive Strength	Water Vapor Permeability
Lower value	5	5	5	0	2	0	2	1
Similar value	2	0	1	0	1	0	0	1
Higher value	3	4	0	1	5	1	0	1

**Table 13:** Performance of lime-based recycled mixed aggregate mortars based on hardened mortar properties, as reported in reviewed studies.

	Study	Binder Type	Mortar Mix	Masonry and Ceramic Waste Specifics and Replacement Ratio	Special Additions
MASONRY MORTAR	Corinaldesi, 2009 [106]	Hydraulic lime	1:3 binder to aggregate ratio by mass	Mixed waste 100% replacement	
	Ferreira et al., 2020 [129]	Pozzolanic Portland cement and CH I hydrated lime (Brazil)	1:1:6 cement to lime to aggregate ratio by volume	Mixed recycled aggregates 25, 50, 75, and 100% replacement	
COATING MORTAR	Ferreira et al., 2019 [130]	Pozzolanic Portland cement and CH I hydrated lime (Brazil)	1:1:6 cement to lime to aggregate ratio by volume	Mixed recycled aggregates 25, 50, 75, and 100% replacement	
	Ferreira et al., 2021 [131]	Pozzolanic Portland cement and CH I hydrated lime (Brazil)	1:1:6, 1:1:7, and 1:2:9 cement to lime to aggregate ratio by volume	Mixed recycled aggregates 100% replacement	
	Mazurana et al., 2022 [132]	CP II F 32 cement and dolomitic lime (Brazil)	1:2:6 cement to lime to aggregate ratio by mass	Mixed CDW 25, 50, 75, and 100% replacement	
	Ruviaro et al., 2022 [133]	Portland-fly ash cement and dolomitic lime (Brazil)	1:2:8 cement:lime:sand by volume	Mixed CDW 30% replacement	
CULTURAL HERITAGE	Stefanidou et al., 2014 [134]	Hydrated lime	1:3 binder to aggregate ratio by mass	Mixed CDW 100% replacement	Superplasticizer

**Table 14:** Performance of lime-based recycled mixed aggregate mortars based on fresh mortar properties, as reported in reviewed studies.

Comparison with Reference Mortars with Natural Aggregates	Fresh Mortar Properties				
	Water Requirement for Similar Consistency	Bulk Density	Air Content	Water Retention	Workable Life
Lower value	0	0	2	0	0
Similar value	0	2	0	1	0
Higher value	7	0	1	1	0

**Table 15:** Performance of lime-based recycled mixed aggregate mortars based on hardened mortar properties, as reported in reviewed studies.

Comparison with Reference Mortars with Natural Aggregates	Hardened Mortar Properties							
	Flexural Strength	Compressive Strength	Bulk Density	Shrinkage	Capillary Water Absorption	Bond Strength	Adhesive Strength	Water Vapor Permeability
Lower value	5	4	3	0	2	0	1	0
Similar value	0	1	1	0	0	0	1	0
Higher value	3	4	0	1	2	2	0	0

## Data Availability

The original contributions presented in the study are included in the article/Supplementary Materials, further inquiries can be directed to the corresponding author.

## Supplementary Materials

Supplementary data are available on Zenodo: https://doi.org/10.5281/zenodo.13120883 (accessed on 29 July 2024). Table S1: Table key; Table S2: Mortar properties’ comparison for cement-based mortars with natural aggregate replacement by recycled concrete aggregates; Table S3: Compressive strengths of cement-based mortars with natural aggregate replacement by recycled concrete aggregates; Figure S1: Compressive strength trend of cement-based mortars with natural aggregate replacement by recycled concrete aggregates; Table S4: Water demand of cement-based mortars with natural aggregate replacement by recycled concrete aggregates; Figure S2: Water demand trend of cement-based mortars with natural aggregate replacement by recycled concrete aggregates; Table S5: Mortar properties’ comparison for cement-based mortars with natural aggregate replacement by recycled masonry aggregates; Table S6: Compressive strengths of cement-based mortars with natural aggregate replacement by recycled masonry aggregates; Figure S3: Compressive strength trend of cement-based mortars with natural aggregate replacement by recycled masonry aggregates; Table S7: Water demand of cement-based mortars with natural aggregate replacement by recycled masonry aggregates; Figure S4: Water demand trend of cement-based mortars with natural aggregate replacement by recycled masonry aggregates; Table S8: Mortar properties’ comparison for cement-based mortars with natural aggregate replacement by recycled mixed aggregates; Table S9: Compressive strengths of cement-based mortars with natural aggregate replacement by recycled mixed aggregates; Figure S5: Compressive strength trend of cement-based mortars with natural aggregate replacement by recycled mixed aggregates; Table S10: Water demand of cement-based mortars with natural aggregate replacement by recycled mixed aggregates; Figure S6: Water demand trend of cement-based mortars with natural aggregate replacement by recycled mixed aggregates; Table S11: Mortar properties’ comparison for lime-based mortars with natural aggregate replacement by recycled masonry aggregates; Table S12: Compressive strengths of lime-based mortars with natural aggregate replacement by recycled masonry aggregates; Figure S7: Compressive strength trend of lime-based mortars with natural aggregate replacement by recycled masonry aggregates; Table S13: Water demand of lime-based mortars with natural aggregate replacement by recycled masonry aggregates; Figure S8: Water demand trend of lime-based mortars with natural aggregate replacement by recycled masonry aggregates; Table S14: Mortar properties’ comparison for lime-based mortars with natural aggregate replacement by recycled mixed aggregates; Table S15: Compressive strengths of lime-based mortars with natural aggregate replacement by recycled mixed aggregates; Figure S9: Compressive strength trend of lime-based mortars with natural aggregate replacement by recycled mixed aggregates; Table S16: Water demand of lime-based mortars with natural aggregate replacement by recycled mixed aggregates; Figure S10: Water demand trend of lime-based mortars with natural aggregate replacement by recycled mixed aggregates.
